# Suicide signaling by GSDMA: a single-molecule mechanism for recognition and defense against SpeB-expressing GAS

**DOI:** 10.1038/s41392-022-01011-0

**Published:** 2022-05-07

**Authors:** Judit Symmank, Collin Jacobs, Ulrike Schulze-Späte

**Affiliations:** 1 Department of Orthodontics, University Hospital Jena, Leutragraben 3, 07743 Jena, Germany; 2 Section of Geriodontics, Department of Conservative Dentistry and Periodontics, University Hospital Jena, An der Alten Post 4, 07743 Jena, Germany

**Keywords:** Infectious diseases, Inflammation, Infection

In a recent article published in *Nature*,^1^ Deng and colleagues uncovered a single-molecule mechanism for protection against the harmful human microbial pathogen *Streptococcus pyrogenes*.

The Gram-positive Group A *Streptococci* (GAS) are responsible for a variety of acute infections ranging from noninvasive, mildly progressive to invasive infectious diseases with high mortality rates. GAS virulence factors thereby condition the invasion of the bloodstream and soft tissue, leading to invasive systemic dissemination.^2^ Deng et al. identified GSDMA not only as a sensor for intracellular streptopain (SpeB), an important GAS virulence factor, but also as an effector protein for host epithelial cell pyroptosis. This programmed cell death mechanism serves to defeat intracellular infection by triggering the elimination of the infected skin cell, thus depriving the pathogen of its protective niche while inducing an inflammatory response. This ultimately prevents bacterial penetration of the epithelial barrier and a subsequent systemic dissemination of the pathogen.^3^

In initial experiments, Deng et al. demonstrated in vivo by subcutaneous infection of different GAS strains into mouse dorsal skin and in vitro infective electroporation of murine keratinocytes that SpeB-sufficient strains induce pyroptotic cell death of epithelial cells. Mutant SpeB strains were unable to induce these degenerative processes, which were evident in severe tissue damage, increased infiltration of neutrophils, elevated lactate dehydrogenase levels, and balloon-like morphology of pyroptotic cells. Such morphological changes are typically caused by caspase 1-dependent cell membrane pore formation, a hallmark of programmed pyroptotic cell death, ultimately followed by water influx and osmotic cell lysis due to disrupted cellular ion gradients.^3^ In addition, the proforms of the pro-inflammatory cytokines IL-1β and IL-18 are cleaved by caspase 1 and released as active forms during pyroptotic cell destruction, further stimulating the protective inflammatory tissue response.

To analyze host genes involved in SpeB-associated cell death, Deng et al. performed whole genome CRISPR screen of Cas9-expressing human epithelial-like A431 cells and identified GSDMA as a promising intracellular candidate. Subsequent *GSDMA* knockout experiments confirmed the crucial role of this gasdermin protein family member in SpeB-induced cell death, as GDSMA-deficient cells no longer exhibited balloon-like morphology or massive LDH release under pathogenic stimulation. The gasdermin protein family consists of several highly conserved members, with GSDMA being primarily expressed in epithelial cells of the skin.^4^ Similar to the other gasdermins, GSDMA also consists of an N-terminal pore-forming domain, a linker, and a C-terminal repression domain. After cleavage by various caspases in the linker region, pore-forming activities were observed for most gasdermins in response to various stimuli. Notably, Deng et al. demonstrated that caspase inhibitors did not induce changes in the induced pyroptosis in GSDMA- and SpeB-cotransfected cells, whereas inhibition of SpeB cysteine protease activity completely prevented proteolytic cell death. Thus, this study postulates a caspase 1-independent pathway of activating pyroptotic processes in epithelial cells. Further, for the activation of this skin cell lytic death program, SpeB needs to be internalized, i.e., present intracellularly.

In contrast to the N- and C-terminal highly conserved regions, the linker region is quite variable in sequence and length among gasdermin family members.^5^ This seems to be important for the activation of specific gasdermins and their regulation by different cysteine and serine proteases when ensuring cell death mechanisms due to diverse pathological stimuli. For GSDMA, unlike all other gasdermins, no eukaryotic proteases have yet been characterized,^5^ therefore making the screening for potential effectors particularly interesting. Deng et al. postulate that the virulence factor SpeB can directly cleave GSDMA. Thus, the authors demonstrated a concentration-dependent cleavage in the GSDMA linker region after Gln246. The catalytically active SpeB was highly selective, thereby not cleaving the highly recombinant GSDMD nor GSDME. In line, other bacterial proteases of a selection of gram-positive and gram-negative bacteria that can cause skin infections (*Mycobacterium tuberculosis, Pseudomonas aeruginosa and Staphylococcus aureus*) tested did not cleave GSDMA, leading the authors to conclude that both molecular components have some specificity for each other in the regulation of pyroptotic cell death.

With further investigations, Deng et al. deciphered a molecular pathway through which SpeB and GSDMA trigger the pyroptosis of infected cells. SpeB-mediated proteolytic cleavage of GSDMA resulted in the release of the N-terminal fragment (GSDMA-NT), its oligomerisation and binding to phosphatidylserine (PS), 3-O-sulfogalactosylceramide and cardiolipin (CL) as shown by a protein–lipid overlay assay. Unlike full-length GSDMA or C-terminal fragments, GSDMA-NT caused liposome leakage, leading the authors to conclude that GSDMA disrupts distinct acidic lipid membranes in a SpeB-dependent manner.

For in vivo validation, the authors subcutaneously infected *Gsdma1*-knockout mice with SpeB. Interestingly, mice express three GSDMA homologs (*Gsdma1*, *Gsdma2* and *Gsdma3*). However, only *Gsdma1* and *Gsdma3* are present in skin with *Gsdma1* possessing the conserved SpeB cleavage site of human GSDMA.^5^ Detailed analysis of *Gsdma1*-deficient mice confirmed that GSDMA is relevant for the pyroptolytic response of epithelial cells to SpeB and even crucial for GAS-immunity of the host. This was demonstrated by the observation that knockout animals did not develop dermonecrotic skin lesions, but showed more severe systemic disseminating infections with increased bacterial loads in liver and spleen, which correlated with significantly increased mortality.

**Fig. 1:**
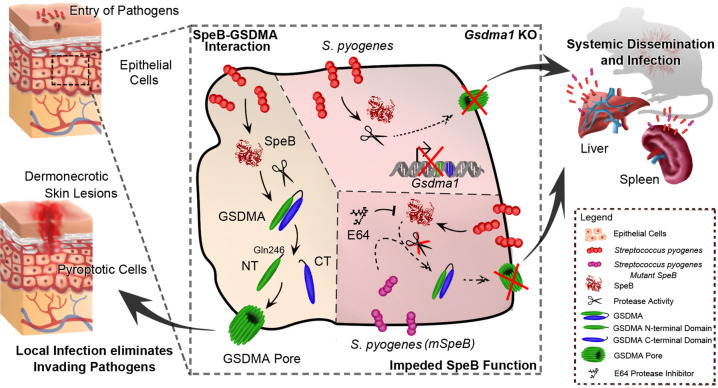
GSDMA-driven pyroptosis of epithelial cells as a single-molecule mechanism for detection and elimination of SpeB-expressing *Streptococcus pyrogenes*. Following pathogen invasion, caspase 1-independent activation of the gasdermin GSDMA occurs via direct proteolytic cleavage by SpeB. Subsequent GSDMA pore formation in the host cell membrane promote pyroptosis of the infected cells, ultimately leading to local inflammation and elimination of the pathogen. *Gsdma1* deficiency and SpeB mutation/inhibition fail to result in activated GSDMA and thus the local immune response, resulting in systemic dissemination and infection of multiple organs. SpeB protein structure was designed by Universal Protein Resource (UniProtKB accession number P0C0J0)

By characterizing a fundamental role of GSDMA and the virulence factor SpeB in GAS infection, Deng et al. have provided extensive evidence for an exciting single-molecule mechanism in pathogenic immune defense at the epithelial cell layer (Fig. 1). The described mechanism seems to be highly selective and relevant, at least in the murine context, for minimizing the risk of an invasive progression of the infection beyond the epithelial barrier. This may be of particular relevance with regard to tightly controlled expression of virulence factors as well as the high impact of the frequent mutation rate in the streptococcal two-component covR/S regulatory system and thus, as also shown in this study, for the evasion of the epithelial host cell immune response by the pathogen.^2^ Since *Gsdma1* is conserved between human and murine species, these findings offer potential opportunities for personalized and targeted therapeutic interventions that could be further investigated in future studies.
